# Evaluating programmatic reactive focal drug administration impact on malaria incidence in northern Senegal: an interrupted time series analysis

**DOI:** 10.1186/s12936-025-05245-5

**Published:** 2025-01-25

**Authors:** Ellen Leah Ferriss, Yakou Dieye, Moustapha Cissé, Gnagna Dieng Sow, Jean Louis Lankia, Damien Diedhiou, Abiboulaye Sall, Tamba Souane, Tidiane Thiam, Doudou Sene, Elhadji Doucouré, Ibrahima Diallo, Adam Bennett, Caterina Guinovart

**Affiliations:** 1 PATH, 2201 Westlake Ave Ste 200, Seattle, WA 98121 USA; 2 PATH, Rue Saint John Perse X F, Dakar, Sénégal; 3 Programme National de Lutte contre le Paludisme, Ministère de la Santé, Rue Aimé Césaire X E, Dakar, Sénégal; 4 Barcelona Institute for Global Health, Hospital Clínic-Universitat de Barcelona, Rosselló, 132, 08036 Barcelona, Spain

**Keywords:** Malaria, Reactive focal drug administration, RFDA, Elimination

## Abstract

**Background:**

The World Health Organization conditionally recommends reactive drug administration to reduce malaria transmission in settings approaching elimination. However, few studies have evaluated the impact of reactive focal drug administration (rFDA) in sub-Saharan Africa, and none have evaluated it under programmatic conditions. In 2016, Senegal’s national malaria control programme introduced rFDA, the presumptive treatment of compound members of a person with confirmed malaria, and reactive mass focal drug administration (rMFDA), an expanded effort including neighbouring compounds during an outbreak, in 10 low transmission districts in the north of the country. This evaluation sought to measure the impact of rFDA and rMFDA on malaria incidence.

**Methods:**

An interrupted time series analysis was conducted with routine surveillance data on health post-level monthly confirmed malaria case counts from the District Health Information Software (DHIS2). The study evaluated the change in incidence following rFDA and rMFDA rollout (level change), which ranged from August 2016 to November 2019, and monthly thereafter (trend change), using an adjusted negative binomial regression model with data from January 2015 through January 2020. The model was used to estimate the number of cases averted via a counterfactual simulation.

**Results:**

No incidence rate reductions were estimated immediately following rollout (level change: incidence rate ratio (IRR) = 1.00, 95% credible interval (CI) = 0.76, 1.33). However, rFDA and rMFDA were associated with a 4% monthly decline in incidence relative to the baseline trend (trend change: IRR = 0.96, 95% CI = 0.95, 0.98). Over the study period, RFDA and rMFDA were estimated to avert 2,070 (95% CI = 577, 4,367) of 4,108 (95% CI = 2,620, 6,425) malaria cases.

**Conclusions:**

RFDA and rMFDA were associated with reduced malaria incidence in northern Senegal, supporting their use in malaria control in very low transmission areas. However, additional strategies are likely needed to achieve elimination in this setting.

**Supplementary Information:**

The online version contains supplementary material available at 10.1186/s12936-025-05245-5.

## Background

The World Health Organization (WHO) recommends reactive drug administration to prevent or reduce malaria in areas approaching elimination, albeit with low certainty evidence [1]. The efficacy of reactive focal drug administration (rFDA), the treatment of all household members and sometimes neighbors of a person with confirmed malaria, in reducing local transmission has been evaluated in a limited number of randomized controlled trials (RCTs), generating mixed evidence. In Namibia, rFDA reduced confirmed malaria incidence by 48% compared to reactive case detection (RACD), the testing and treatment of rapid diagnostic test (RDT)-positive individuals around an index case [2]. In the Gambia, rFDA reduced the odds of infection by 49%, compared to reactive testing and treatment of symptomatic index case compound members, after restricting to areas that reported malaria during the intervention period [3]. In contrast, rFDA was not associated with lower malaria incidence compared to RACD in Eswatini or Zambia, though a 37% reduction in short-term serological markers of infection was estimated in Zambia [4, 5]. No studies have evaluated routine programmatic implementation of rFDA in a *Plasmodium falciparum-*endemic area.

In 2016, Senegal’s national malaria control programme, the Programme National de Lutte contre le Paludisme (PNLP), introduced rFDA and reactive mass focal drug administration (rMFDA), the presumptive treatment of larger transmission foci, in the north of the country. This study estimated the impact of programmatic implementation of rFDA and rMFDA on confirmed malaria incidence from 2016 to 2020.

## Methods

### Study design

A quasi-experimental design was used to evaluate the change in confirmed malaria incidence following rFDA and rMFDA introduction using an interrupted time series (ITS) analysis. The interruption was set at the time of roll out for each health post, allowing us to estimate rFDA and rMFDA impact by attributing any change in the malaria incidence level or trend to these interventions, after adjusting for contemporaneous malaria interventions, climatic patterns, and other potential confounders. This evaluation used health-post level routine surveillance data on monthly confirmed malaria case counts from January 2015 through January 2020. Only health posts with at least 80% rFDA/rMFDA coverage were included in this analysis in order to estimate intervention impact under optimal programmatic implementation, assuming rFDA and rMFDA would be less effective at lower coverage levels.

### Study area

Malaria transmission in northern Senegal is low and seasonal, with an average annual incidence of under 10 cases per 1,000 population. Malaria is predominantly caused by *P. falciparum* and transmitted by the vector *Anopheles arabiensis* [6]. Interventions include case management, intermittent preventive treatment in pregnancy, and long-lasting insecticidal net (LLIN) distributions through routine channels and universal campaigns, which occurred in June, 2016, and July and August, 2019.

**Fig. 1:**
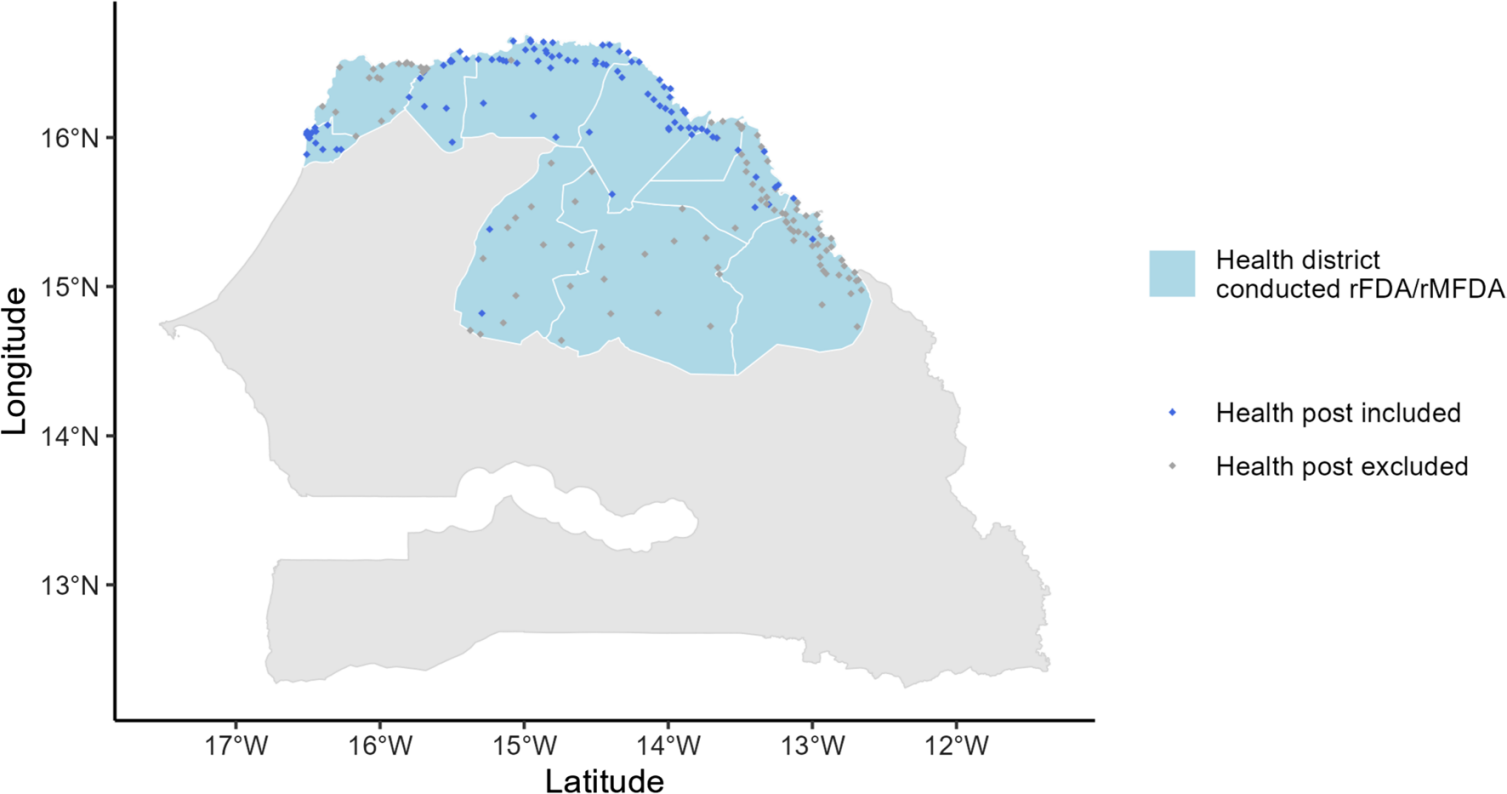
Health posts in districts conducting rFDA and rMFDA. Only those that achieved at least 80% intervention coverage (blue dots) were included in the analysis**.** Health posts with coverage below 80% or in a health district with predominantly non-local transmission and that previously conducted RACD and rFDA were excluded (gray dots)

**Fig. 2:**
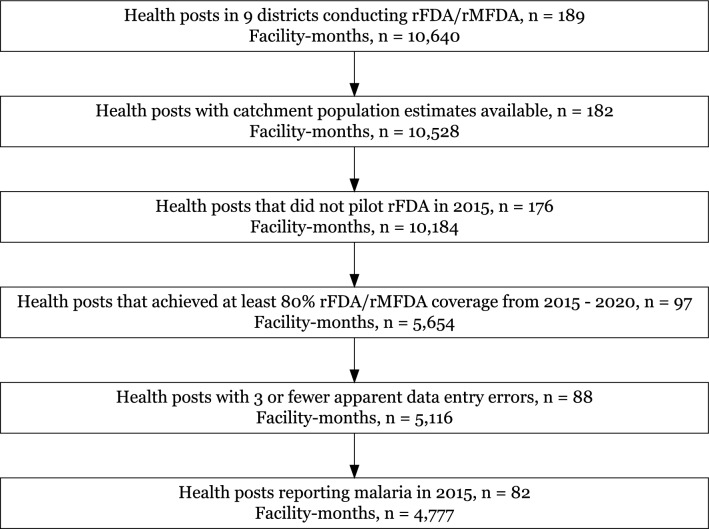
Diagram of data exclusion showing the number of health posts and non-missing observations. Data from one health district that was excluded a priori due to predominantly non-local transmission and previous reactive interventions are omitted

From August 2016 to January 2017, Senegal added rFDA and rMFDA to its malaria control programming in 10 districts in the north, the lowest transmission part of the country, to further reduce malaria. 82 health posts with high reported rFDA and rMFDA coverage (≥80%) through 2020 were included in this analysis (Figs. 1 and 2). Included facilities served 780,000 people across 8 districts. One health district with predominantly non-local transmission and where RACD and rFDA had previously been conducted was not considered in this study [7].

### Intervention

Individuals who presented to a health facility or community health worker for malaria care and received a confirmed malaria diagnosis were considered index cases; treated with artesunate/amodiaquine (AS/AQ), artemether-lumefantrine (AL), or dihydroartemisinin-piperaquine (DHAP), in addition to single low dose primaquine (non-pregnant individuals aged 10 years and older); and administered a standardized questionnaire capturing details of their infection and travel history. RFDA was triggered in each index case’s compound(s) if they lived in or had spent at least 1 night in a participating health zone in the past 15 days. Residents of the index case’s compound were visited within 72 h, administered a standardized compound investigation questionnaire, and consenting and eligible residents older than two months given AL (2–5 months of age) or DHAP (≥6 months). In addition, when five or more index cases living within 100-to-150-m of each other were identified within 15 days or three or more cases within 7 days, reactive drug administration was carried out in all compounds within 100-m of each index case (rMFDA).

### Data sources and management

#### Outcome

Monthly confirmed malaria case incidence was calculated for each public health post, i.e., outpatient facility responsible for rFDA/rMFDA, using the number of patients with RDT-confirmed malaria and the annual catchment population. Data were obtained from the District Health Information Software (DHIS2). Facilities reported case data into the system monthly, except during the 2018 data strike, when data were electronically withheld and reported retroactively following the end of the strike.

Monthly case counts (median = 0, quartile 1–quartile 3 (Q1–Q3) = 0–1) were flagged as probable data entry errors and set to missing if they were at least 10 times the monthly median during the quarter of observation for a given facility (probable errors among facilities included in the analysis: n = 38). For example, the number of cases observed in January 2015 was compared against the median of all observations occurring in January, February, and March, 2015 through 2020, at that facility. Facilities were excluded from the analysis if they had four or more presumed errors out of the follow-up period (median follow-up was 60 months) or atypical counts outside of the transmission season, i.e., cases equaled or exceeded typical peak transmission season values for several consecutive months during low transmission season (Fig. 2). Facilities with missing catchment population estimates; those reporting no patients with confirmed malaria in 2015 (pre-intervention); those conducting rFDA/rMFDA at less than 80% estimated coverage; and those that piloted rFDA in 2015 were additionally excluded.

#### Exposure

Exposure to the intervention was modelled as an indicator variable that captured whether a health post had begun conducting rFDA and/or rMFDA. The pre-intervention period was defined as January 2015 through the month prior to rFDA/rMFDA roll-out (the interruption), which ranged from August 2016 through November 2019 by health post. The intervention period was defined as the first month of rFDA/rMFDA at each health post through January 2020.

Health post intervention coverage was used to determine facility inclusion and was estimated as follows. Programmatic index case data and compound investigation data were stripped of personal identifiers and linked using randomly generated keys that replaced the index case unique identifiers. Data for each health post catchment area were aggregated across the period to obtain the proportion of eligible compounds that received the intervention, the number of individuals living in those compounds, and the number receiving treatment. Coverage was estimated as the percentage of eligible index case compounds that were successfully visited for rFDA/rMFDA multiplied by the percentage of compound members treated over the course of the study for each health post, with one coverage estimate calculated per facility. Beginning in October 2018, reporting of rFDA and rMFDA in higher transmission areas declined due to the data strike, leading to drops in estimated intervention coverage and exclusion of affected facilities.

#### Confounders

Monthly outpatient visits were included in the model to adjust for changes in malaria incidence arising from changes in care seeking, rather than changes in transmission. Data on monthly outpatient visits were obtained for each health post from DHIS2, divided by the catchment area population to obtain a care seeking rate, and log-transformed (Supplement Fig. 1).

Models were adjusted for LLIN access, which increased during the rFDA/rMFDA intervention period due to mass campaigns in 2016 and 2019, likely reducing incidence. The number of LLINs distributed during universal campaigns was obtained from PNLP national reports, health district reports, and DHIS2. Health post-level data on LLINs distributed were available in 2019. Otherwise, district-level data were used to estimate health post distributions, with the number of nets proportional to the health post catchment population. The number of available nets was assumed to decrease 5.6% per month following distribution, consistent with a lifespan of 1.5 years [8]. LLIN access was calculated as total nets available divided by the catchment population, scaled by a factor of two, assuming each net was shared by two individuals (Supplement Fig. 2). Estimated LLIN access exceeding 100% was rounded down to 100%.

Urbanicity was also considered a confounder, as urban environments were assumed to have lower malaria risk due to differences in housing construction methods, occupational exposures, and/or vector habitat availability. Health posts were classified as urban or rural using GRID3 gridded population estimates with the PATHtools R package (Supplement Fig. 3) [9, 10]. Health post catchment areas were labeled urban if over 50% of the area within a 2.5 km radius from the health post had a population density of at least 300 people/km^2^ and fell within a contiguous area of at least 10,000 people [11, 12].

Monthly rainfall, nighttime and daytime land surface temperature, enhanced vegetation index (EVI), and month were included to account for environmental changes over the study period that likely influenced vector reproduction and longevity (Supplement Figs. 4–6). 0.05 degree resolution total monthly rainfall from Climate Hazards Group InfraRed Precipitation with Station data (CHIRPS), 6-km resolution average monthly nighttime and daytime land surface temperature from Moderate Resolution Imaging Spectroradiometer (MODIS), and 1-km resolution monthly EVI from Terra MODIS Vegetation Indices were extracted at 0, 1, and 2-month lags and averaged across each health post catchment area [13–16]. Where a monthly estimate was unavailable, the district average for the month was assigned. The best fitting lagged covariates were selected for inclusion in the final model using the Watanabe-Akaike information criterion (WAIC).

The percentage of documented index cases that reported travelling outside the district during the last 60 days, i.e., the travel rate, was obtained for each health post catchment area (Supplement Fig. 7). Travel rates were assumed to capture the degree of importation, either from other districts within Senegal or from other countries, and to be the same for the intervention period and pre-intervention period, during which time data were unavailable.

### Statistical analysis

An ITS model, the basic form of which is shown in Supplement Eq. 1, was conducted to evaluate the impact of rFDA and rMFDA from rollout through January 2020 [24]. An indicator variable was used to estimate the incidence rate change in health post catchment areas in the period immediately following intervention rollout relative to the pre-intervention period (level change). In addition, an interaction term between the pre-intervention time trend and the intervention period captured the monthly change in incidence following rFDA rollout relative to baseline (trend change).

A negative binomial regression model was fit with uninformative priors using the R INLA package (www.r-inla.org) [17–19]. Monthly health post case counts were specified as the outcome and logged health post catchment populations as the offset. Random intercepts for health post catchment area, month, and their interaction, modelled using conditional autoregressive Besag-York-Mollié, autoregressive-1, and independently and identically distributed correlation structures, respectively, were included based on model fit (Supplement Table 1) [20]. Models were adjusted for total outpatient visits, LLIN access, urbanicity, sine and cosine terms capturing seasonality, lagged environmental covariates, and the travel rate, a priori. Missing outcomes were predicted by the model. An additional model without exposure terms was estimated to review the impact of possible collinearity between rFDA and rMFDA and potential confounders.

The number of clinical cases averted by rFDA and rMFDA was estimated via counterfactual simulation. The main model was used to estimate the number of people with confirmed malaria under a hypothetical scenario with no intervention and, separately, given rFDA/rMFDA. The number of cases averted was taken as the difference. Credible intervals were generated by sampling 1,000 new estimates for the regression coefficients from the posterior distribution, recalculating cases averted for each sample, and taking the 2.5th and 97.5th quantiles. All statistical analysis was conducted in R version 4.3.1.

## Results

From August 2016 to January 2020, 15,712 index cases were eligible for rFDA or rMFDA across nine districts at 210 health posts, including those not included in this evaluation due to low reported intervention coverage. 168,180 individuals from 9,028 compounds were treated through rFDA and 22,294 individuals from 1,238 compounds through rMFDA (Table 1). The percentage of eligible compounds that were reached following index case detection ranged from 59 to 99% by district, and the percentage of visited compound members who received treatment ranged from 88 to 96%. Median time from index case documentation to rFDA was three or fewer days in six of nine health districts (range: 1–7 days). Estimated district intervention coverage ranged from 56 to 93% from August 2016 through January 2020, with substantial decreases in higher transmission districts over the study period. District coverage ranged from 84 to 92% in 2017, the first full year of rFDA, down to 32–92% in 2019 (Supplement Fig. 8).

**Table 1:** Index case and neighbouring compounds eligible for follow-up that received rFDA, rMFDA, or neither intervention

Health district	Compounds eligible for follow-up	rFDA, n (%)*	rMFDA, n (%)*	No intervention received, n (%)*
Dagana	142	137 (96.5)	3 (2.1)	2 (1.4)
Kanel	4957	2293 (46.3)	644 (13.0)	2020 (40.8)
Linguère	2106	1549 (73.6)	0 (0.0)	557 (26.4)
Matam	891	633 (71.0)	0 (0.0)	258 (29.0)
Pété	548	504 (92.0)	32 (5.8)	12 (2.2)
Podor	400	352 (88.0)	42 (10.5)	6 (1.5)
Ranérou	4331	2249 (51.9)	492 (11.4)	1590 (36.7)
Saint-Louis	1064	978 (91.9)	25 (2.3)	61 (5.7)
Thilogne	394	333 (84.5)	0 (0.0)	61 (15.5)

^*^Percent totals may not equal 100% due to rounding

**Fig. 3:**
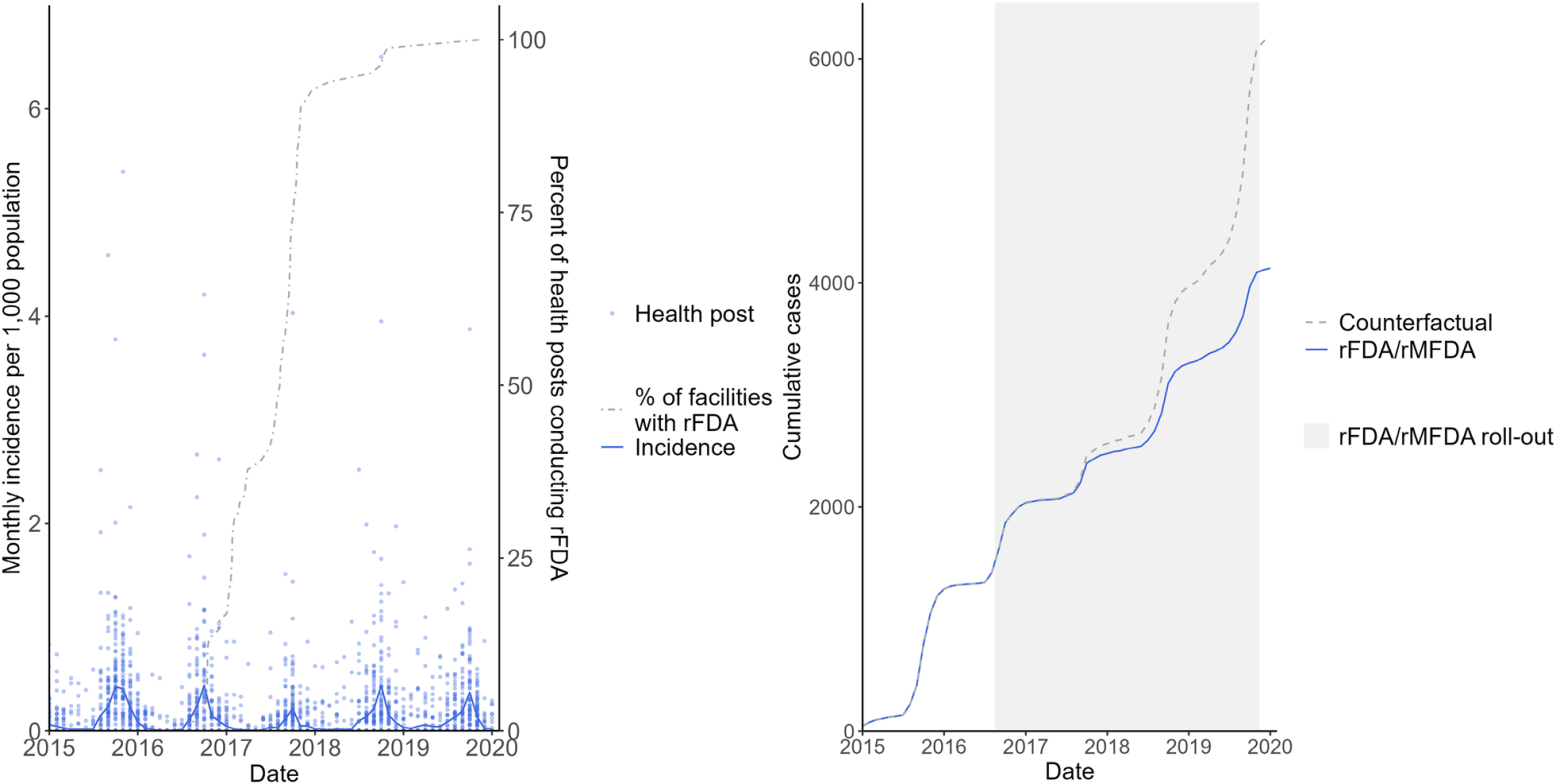
Monthly incidence (left) and modelled cumulative cases (right) shown with intervention rollout. Monthly incidence is shown in blue across facilities (line) and by facility (point) against the percentage of health posts conducting rFDA/rMFDA with high coverage, i.e. ≥80% (left). The estimated cumulative number of people with confirmed malaria across health posts with high rFDA/rMFDA coverage is shown in blue with the counterfactual (no rFDA/rMFDA) in gray (right)

Health posts included in the study contributed 5,002 facility-months, 225 of which were missing outcomes. Study area-wide incidence decreased from 1.6 cases per 1000 population (annual parasite index (API)) in 2015, prior to rFDA and rMFDA rollout, to 1.1 API in 2019, a 31% reduction (Fig. 3). However, incidence during the higher transmission months, July through January, decreased only 17% from the pre-intervention to the intervention period across years. Median facility-level rFDA and rMFDA coverage was 90% (Q1–Q3 = 87–93%) over the study period, with relatively little interannual variation (first full year, 2017: median = 92%, Q1–Q3 = 85–95%; final full year, 2019: median = 90%, Q1–Q3 = 83–93%). The percentage of index cases in each health post catchment area that reported travel outside the district in the past 60 days averaged 59% and ranged from 3 to 100%. Between the pre-intervention and intervention periods, rainfall slightly decreased, while temperatures increased. Further, LLIN access increased during the intervention period due to mass distribution campaigns in 2016 and 2019, while outpatient visits declined (Table 2).

**Table 2:** Health post characteristics by period across facility-months

Covariates	Pre-intervention period N = 2500	Intervention period N = 2502
Monthly total rainfall (Jun–Oct), mm**	37 (10, 86)	33 (12, 68)
Monthly EVI**	0.13 (0.12, 0.16)	0.13 (0.13, 0.15)
Monthly daytime temperature, °C**	35.4 (32.9, 39.0)	36.1 (33.2, 39.2)
Outpatient visits per 1,000 population**	45 (30, 65)	37 (24, 55)
Urban, facility-months*	386 (15%)	407 (16%)
LLIN access, %**	7 (4, 70)	30 (19, 59)

^*^n (%) **median (Q1, Q3)

While no initial drop in incidence was estimated following rFDA and rMFDA introduction (level change: incidence rate ratio (IRR) 1.00, 95% credible interval (CI) = 0.76, 1.33), incidence rates were estimated to decline 4% more each month following roll-out compared to the pre-intervention trend (trend change: IRR = 0.96, 95% CI = 0.95, 0.98) (Table 3). Over the duration of the study, rFDA and rMFDA were estimated to avert 2,070 (95% CI = 577; 4,367) of 4,108 (95% CI = 2,620; 6,425) malaria cases in health facility catchment areas with at least 80% intervention coverage. Modelled impact was nearly absent until the 2017 transmission season, consistent with rolling rFDA/rMFDA introduction that largely occurred that year (Fig. 3).

**Table 3:** Adjusted incidence rate ratios estimating rFDA and rMFDA impact

Variables	IRR	95% CI
Months since study start	1.02	0.99, 1.05
Level change: Intervention/pre-intervention period	1.00	0.76, 1.33
Trend change: Months since rFDA roll-out (Months since study start × Intervention/pre-intervention period)	0.96	0.95, 0.98
Sine term (seasonality)	0.36	0.22, 0.57
Cosine term (seasonality)	1.27	0.84, 1.91
EVI, 0.1-unit increase	1.53	1.33, 1.77
Day temperature (2-month lag)	0.95	0.90, 0.99
Rainfall (2-month lag), 10-mm increase	1.07	1.03, 1.11
Outpatient visits per population, log-unit increase	1.73	1.50, 2.00
Urban	0.94	0.51, 1.72
LLIN access, 10% increase	0.93	0.86, 1.00
Index cases reporting travel outside the district, 10% increase	0.91	0.85, 0.98

*IRR* incidence rate ratio, *CI* credible interval

## Discussion

In northern Senegal, rFDA and rMFDA were associated with a 4% monthly reduction in confirmed malaria incidence compared to the pre-intervention trend, suggesting sustained reductions in transmission. While a 50% decrease in cases attributable to rFDA was estimated over the duration of the study, the increasing adjusted pre-intervention trend in incidence might not have continued through 2020 absent the intervention. Thus, this study’s counterfactual scenario likely over-estimated the number of cases that would have been observed without rFDA and rMFDA and, by extension, the number of cases these interventions averted. Nevertheless, the findings are similar to those from RCTs in Namibia and the Gambia, areas with comparable incidence to northern Senegal and high intervention coverage. In Namibia, rFDA reduced incidence by 48% compared to RACD [2]. In the Gambia, the odds of infection were 49% lower in areas that conducted rFDA, compared to screen, test, and treat, after restricting to villages that reported incident malaria, though a non-significant 29% reduction was estimated overall [3].

Greater impact on *P. vivax* malaria has been observed in Peru, where transmission was interrupted following focal drug administration [21]. In contrast, rFDA did not reduce incidence or parasite prevalence compared to RACD in southern Zambia or Eswatini, though a 37% reduction in short-term serological markers of infection in the former show the potential for reduced transmission not captured via routine surveillance [4, 5]. In Eswatini, low intervention coverage and high rates of importation likely reduced rFDA impact, a possibility in this area of northern Senegal, where high travel rates among individuals with malaria were recorded [4]. In areas with substantial transmission away from the home, programmes should seek to identify index case contacts, such as co-travellers or coworkers, who are at increased risk of infection due to shared exposure histories and may additionally benefit from the intervention.

This study supports rFDA for malaria reduction in areas with very low, seasonal transmission and is the first of which the authors are aware to suggest impact under programmatic conditions in a *P. falciparum*-endemic country. Unlike other studies of rFDA in sub-Saharan Africa, this evaluation used routine surveillance data with a quasi-experimental study design. Such designs have significant potential to generate evidence for malaria control programmes using existing data to support national strategic planning [22, 23]. However, they are potentially limited by data quality and resolution, in addition to residual confounding, resulting in less certain estimates of intervention impact.

The findings of this study were likely influenced by programmatic and routine surveillance data quality. DHIS2 monthly case counts did not match the number of index cases documented for rFDA, indicating intervention coverage and malaria incidence estimates were approximate. The median discrepancy between the two datasets was 1 case per facility-month (Q1–Q3 = 0–2 cases per facility-month). Given the low prevailing incidence rates, even these small errors may have led to substantial misestimation of true incidence. Further, non-reporting of rFDA and rMFDA during the 2018 data strike likely resulted in coverage underestimation and subsequent exclusion of facilities that achieved at least 80% coverage. In addition to changes in reporting, changes in treatment seeking may have biased results. These data quality issues may have reduced the precision, and possibly magnitude, of this study’s impact estimates.

Residual confounding may also have resulted in under- or overestimation of rFDA and rMFDA impact. Heterogeneity in LLIN access between health posts within districts was not modelled prior to 2019 and presumably affected incidence. In addition, conditions were more conducive to malaria transmission prior to rFDA and rMFDA introduction. Rainfall and EVI, which were positively associated with malaria risk, were higher during the pre-intervention period, while daytime temperature, which was negatively associated with risk, was lower. Further, the 2016 LLIN mass campaign and rFDA roll-out were similarly timed in some health posts. If environmental covariates and LLIN access were collinear with the trend change, their impact on malaria incidence may have been misattributed to rFDA and rMFDA. To evaluate collinearity, the model was re-fit excluding covariates that captured rFDA and rMFDA. Virtually no change in the impact of environmental covariates or LLIN access on malaria incidence was estimated after excluding exposure variables (not shown). Still, unevaluated external factors may have reduced malaria over time, leading the authors to misattribute declining incidence to rFDA and rMFDA. Given the absence of areas with similar transmission intensity and malaria control programming, contemporaneous incidence trends in areas without rFDA and rMFDA could not be evaluated as a counterfactual.

## Conclusions

This study supports the implementation of rFDA for malaria control in this low transmission area of northern Senegal and bolsters the current WHO recommendation for rFDA in areas approaching elimination. Programme evaluations using routine surveillance data are increasingly important for determining intervention effectiveness in resource constrained environments to inform policy. However, ongoing improvements to routine data quality, which may be pursued through enhanced training, supervision and data monitoring, and possibly routine data quality audits, are needed. As countries pilot rFDA, they may include impact evaluations in implementation planning to ensure rFDA can be assessed for where and when it is most beneficial, helping to build the evidence base for programmes globally.

## Supplementary Information

Additional file 1.

## Data Availability

All data belong to the Senegal Programme National de Lutte contre le Paludisme. Data are available from the corresponding author to individuals who have been granted permission by the PNLP.

## Abbreviations

AL: Artemether-lumefantrine
API: Annual parasite index
CI: Credible interval
CHIRPS: Climate Hazards Group InfraRed Precipitation with Station data
DHAP: Dihydroartemisinin-piperaquine
DHIS2: District Health Information Software
EVI: Enhanced vegetation index
IRR: Incidence rate ratio
ITS: Interrupted time series
LLIN: Long-lasting insecticidal net
MODIS: Moderate Resolution Imaging Spectroradiometer
PNLP: Programme National de Lutte contre le Paludisme
Q1: Quartile 1
Q3: Quartile 3
RCT: Randomized controlled trial
RDT: Rapid diagnostic test
rFDA: Reactive focal drug administration
rMFDA: Reactive mass focal drug administration
WAIC: Watanabe-Akaike information criterion
WHO: World Health Organization
