# Supplementary material for: Evaluating programmatic reactive focal drug administration impact on malaria incidence in northern Senegal: an interrupted time series analysis
Source: Malar J. 2025 Jan 25;24:27. doi: 10.1186/s12936-025-05245-5 (PMC11762883; doi:10.1186/s12936-025-05245-5)
Supplement: Supplementary file 1 — Additional file 1. [file 12936_2025_5245_MOESM1_ESM.docx]

**Supplement**


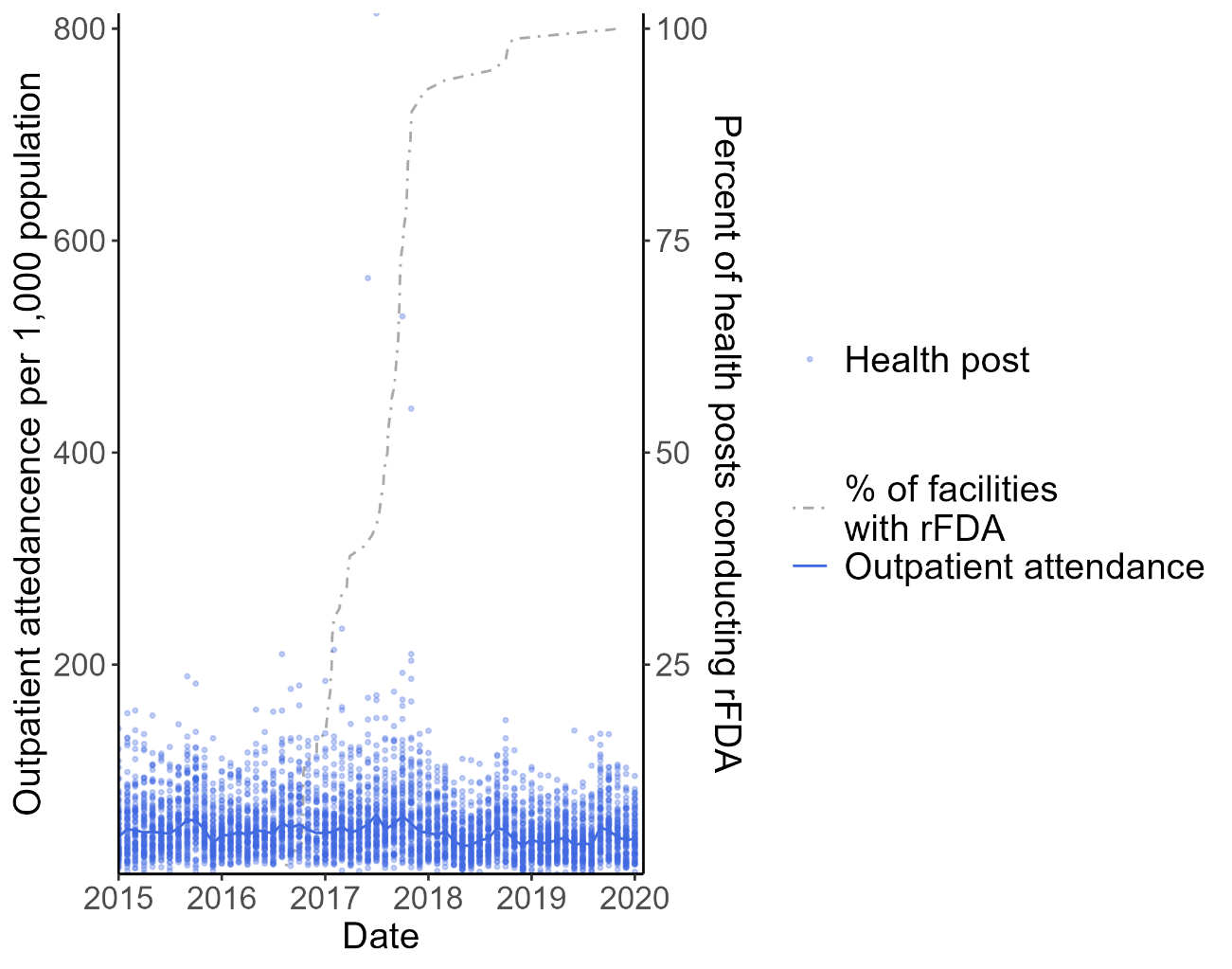


**Supplement Figure 1. Monthly outpatient attendance against the percentage of study health posts conducting rFDA/rMFDA.** Monthly outpatient attendance per 1,000 population is shown for each health post (blue dot) and totaled across health-posts (blue line).


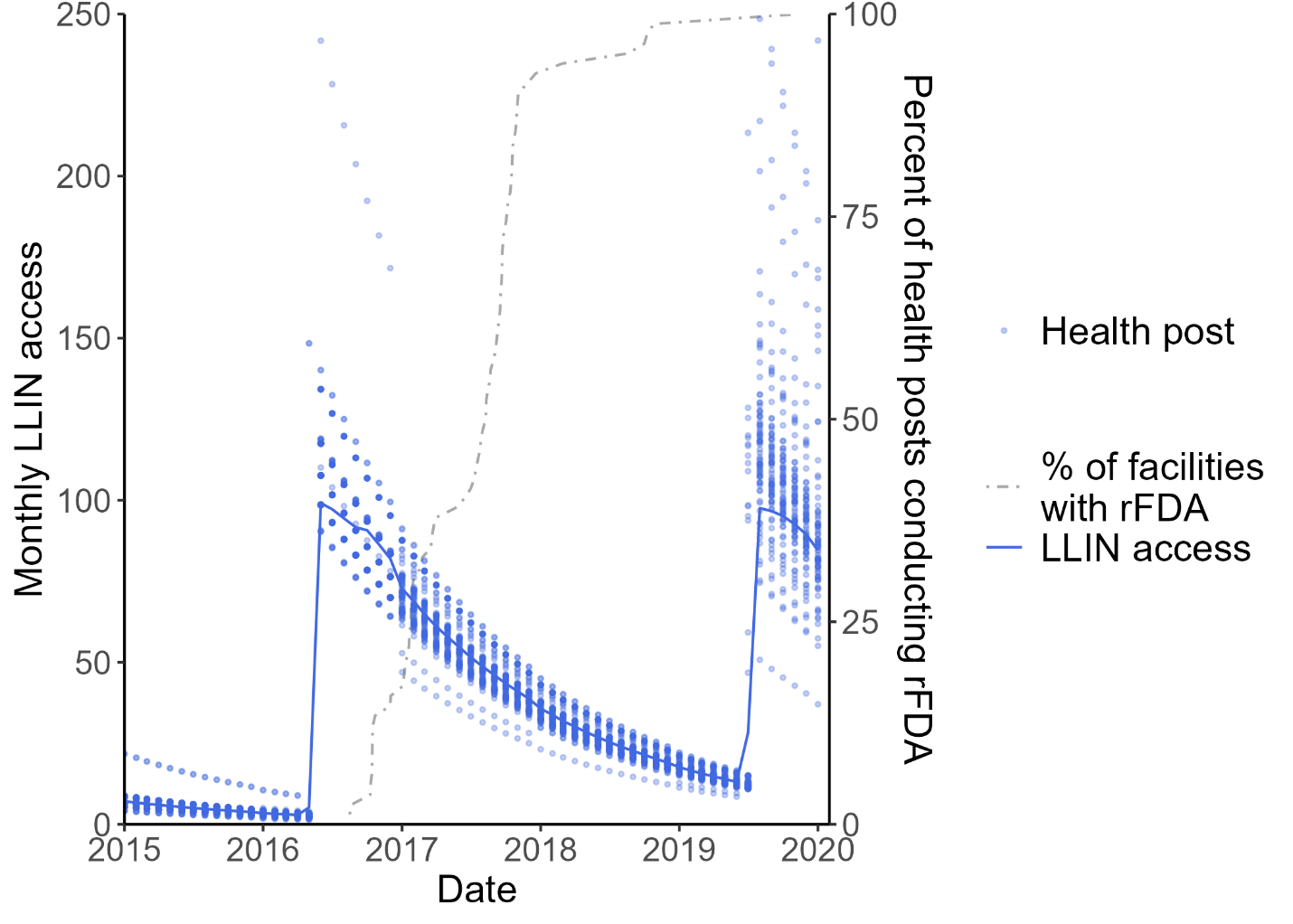


**Supplement Figure 2. Monthly LLIN access against the percentage of study health posts conducting rFDA/rMFDA.** Monthly LLIN access (nets distributed per 2 people, assuming a 5.6% monthly decay) is shown for each health post (blue dot) and averaged across health-posts (blue line).

**
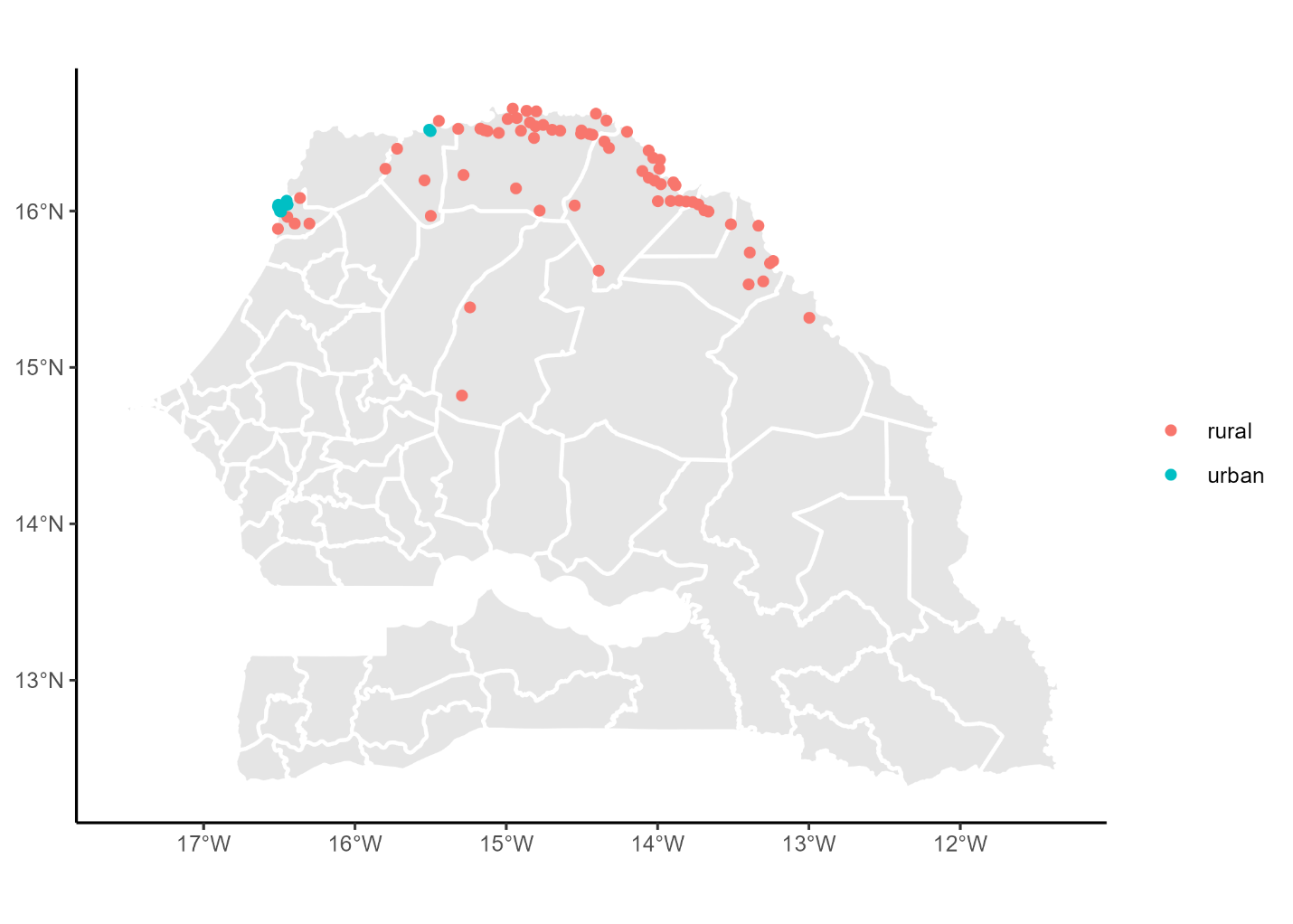
**

**Supplement Figure 3. Study health posts’ urban/rural classification**

**
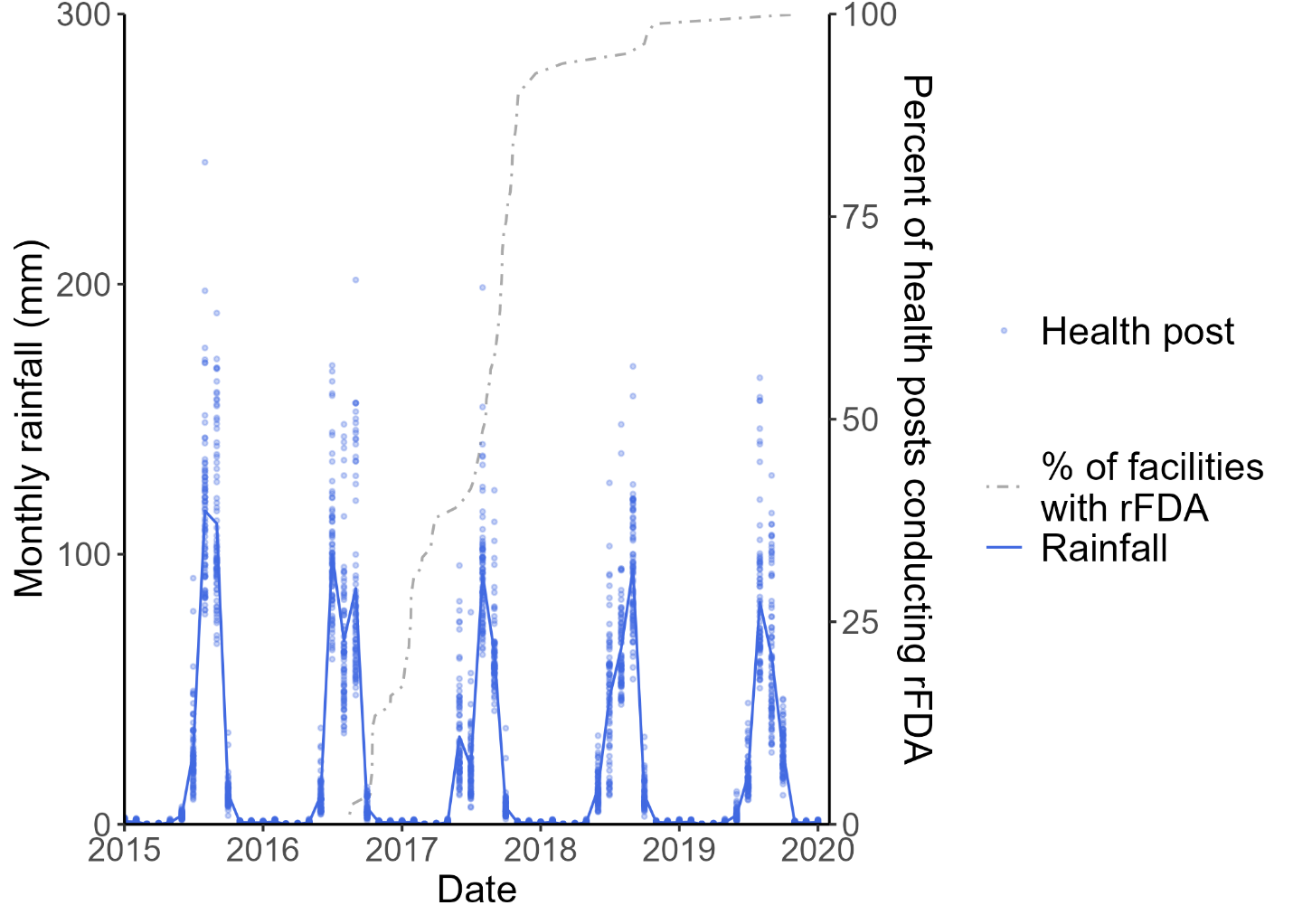
**

**Supplement Figure 4. Monthly rainfall against the percentage of study health posts conducting rFDA/rMFDA.** Monthly rainfall is shown for each health post (blue dot) and averaged across health-posts (blue line).

**
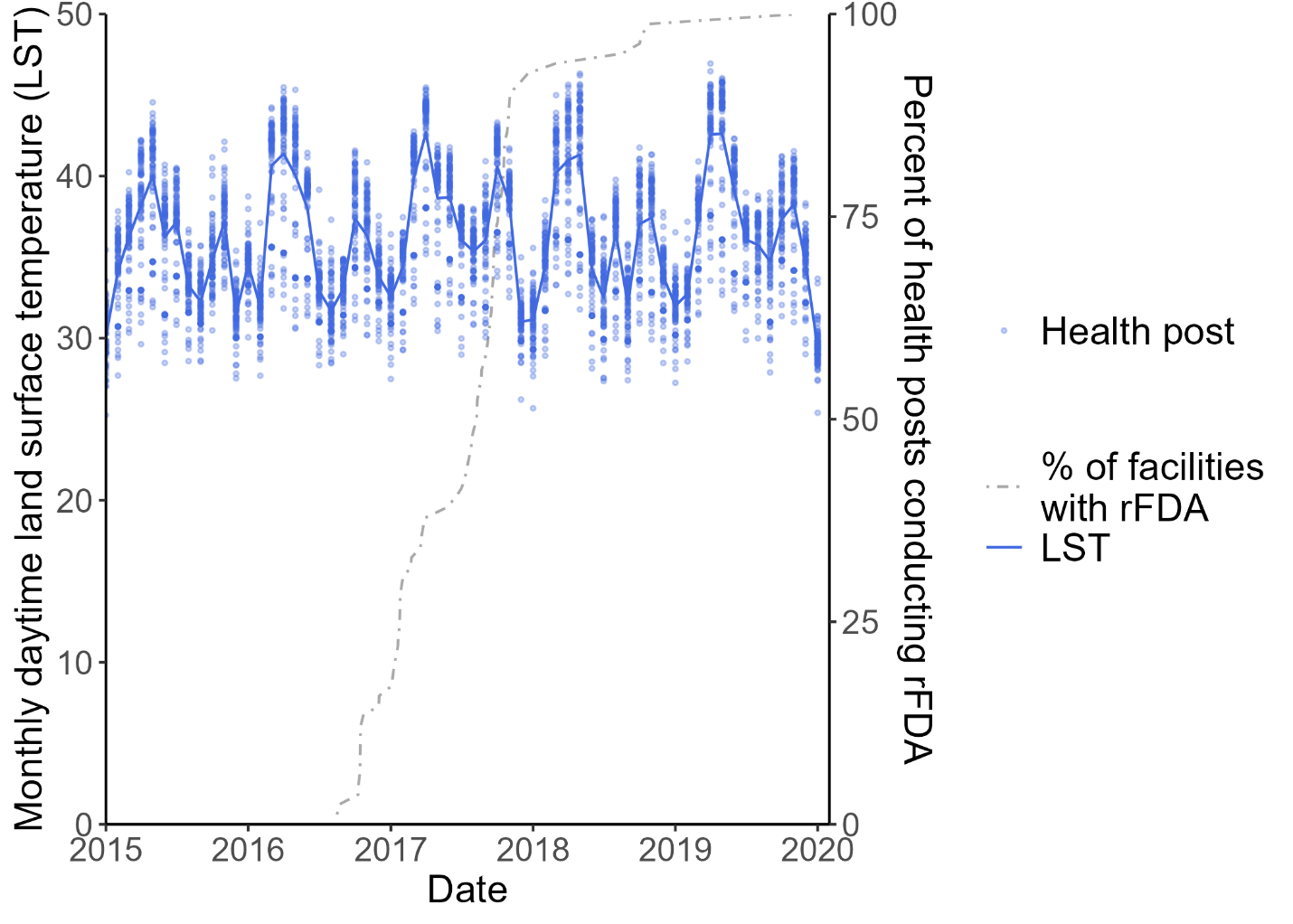
**

**Supplement Figure 5. Monthly daytime land surface temperature (LST) against the percentage of study health posts conducting rFDA/rMFDA.** Monthly daytime LST is shown for each health post (blue dot) and averaged across health-posts (blue line).

**
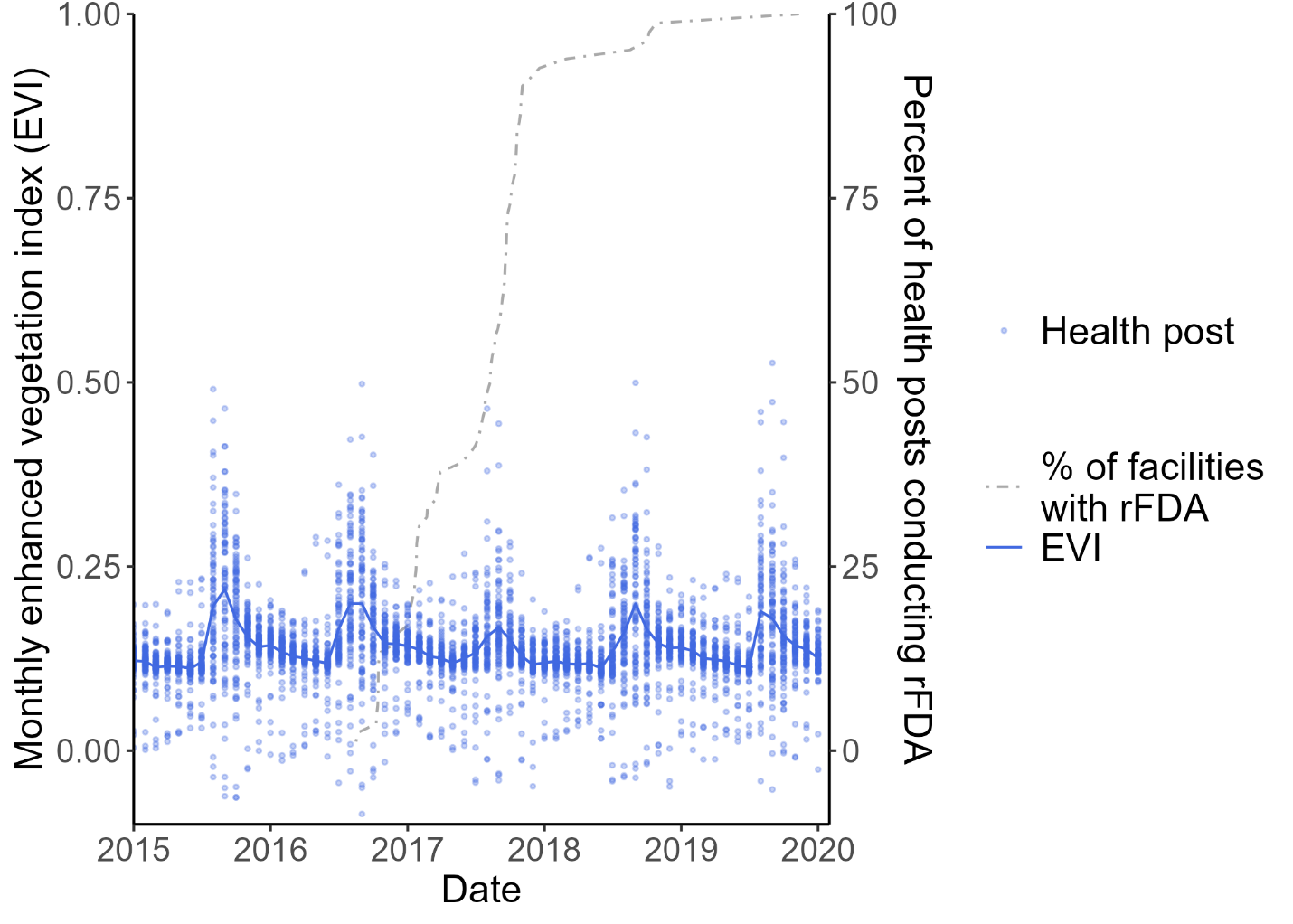
**

**Supplement Figure 6. Monthly enhanced vegetation index (EVI) against the percentage of study health posts conducting rFDA/rMFDA.** Monthly EVI is shown for each health post (blue dot) and averaged across health-posts (blue line).

**
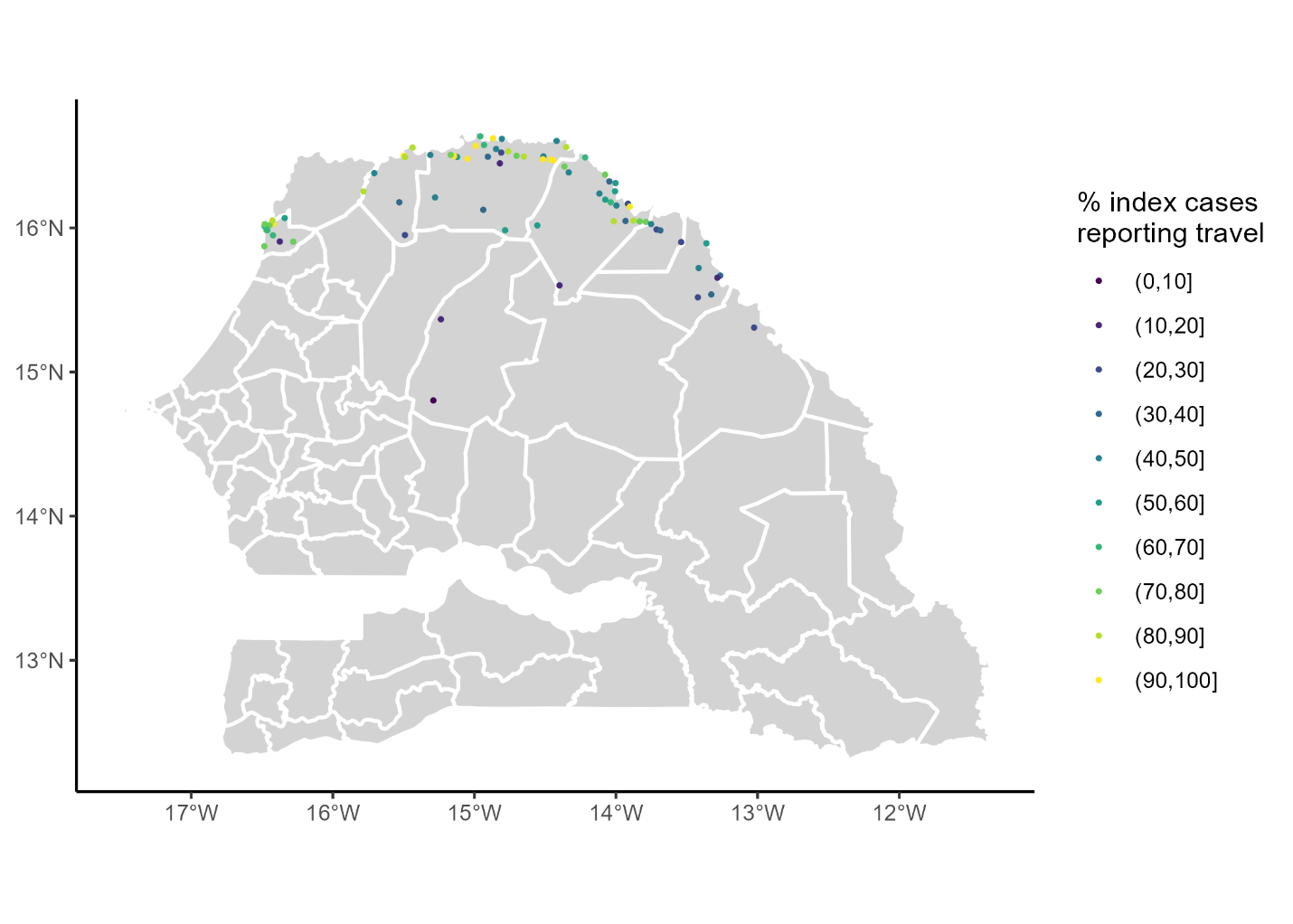
**

**Supplement Figure 7. Study health posts’ travel rate.** Travel rate was defined as the percentage of index cases who traveled outside the district in the past 60 days.

**Supplement Equation 1.**

*log Y_t_ = β_0_ + β_1_T_t_ + β_2_X_t_ + β_3_X_t_T_t_ + log population_t_ + ϵ_t_*

Where Y_t_ represents the number of cases estimated during month t; β_0_ the intercept; β_1_ the pre-intervention incidence time trend; T_t_ the number of months since the beginning of the study; β_2_ the change in incidence immediately after rFDA roll-out (level change); X_t_ an indicator variable denoting the intervention period; β_3_ the relative change in the incidence trend between the intervention and pre-intervention periods (trend change); and population_t_ the health facility catchment area population at month t [29]*.*

**Supplement Table 1. Summary of Watanabe-Akaike information criterion (WAIC) for fully adjusted regression models**

| Random intercepts included in the model (correlation structure) | WAIC |
| --- | --- |
| Month (autoregressive-1) | 8582.26 |
| Month (autoregressive-1) and health post (Besag-York-Mollié) | 8249.37 |
| Month (autoregressive-1), health post (Besag-York-Mollié), and their interaction (independently and identically distributed) | 8224.87 |


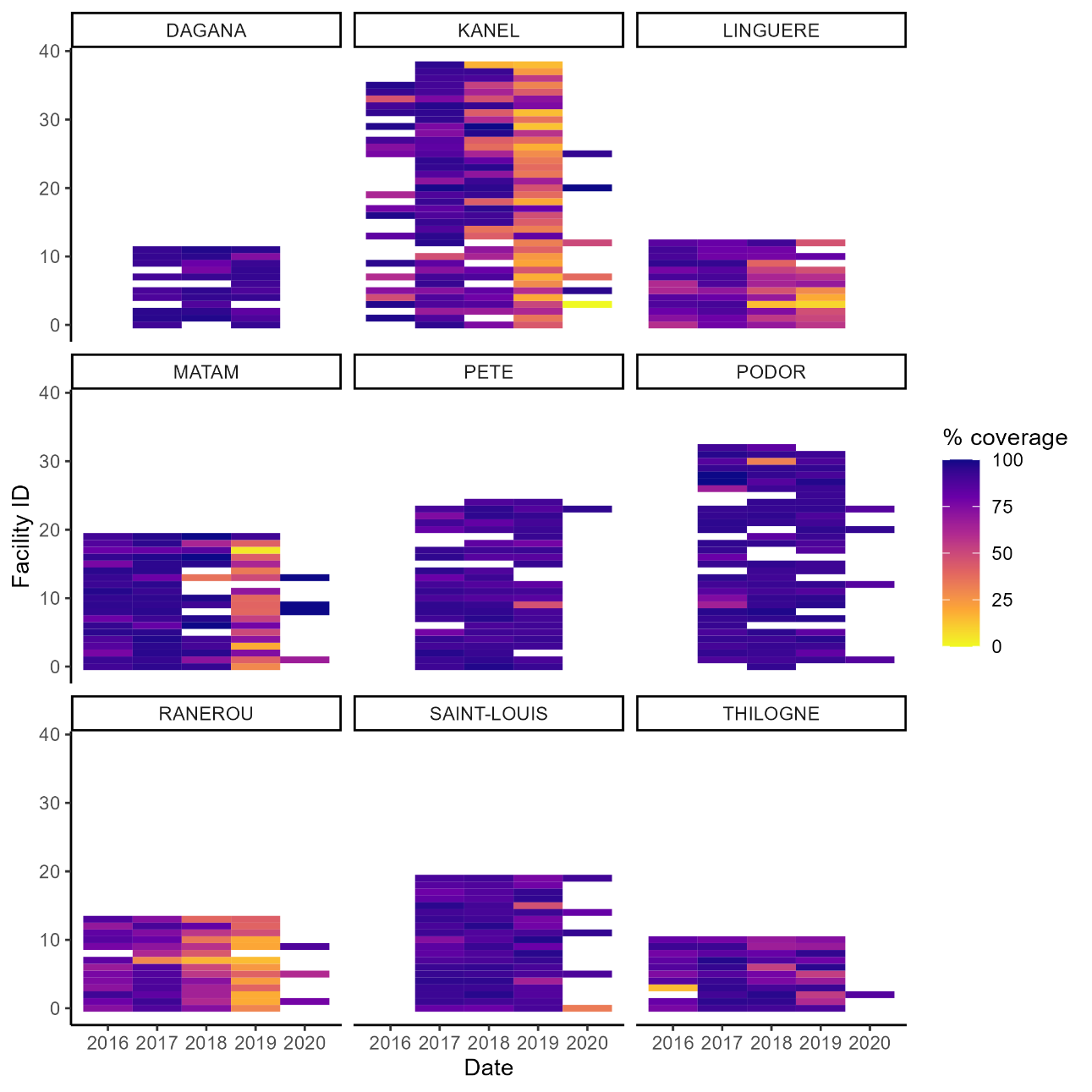


**Supplement Figure 8. Annual rFDA and rMFDA coverage by health district.** Annual rFDA and rMFDA coverage (cell) is shown for each health post (row). Health districts in Saint-Louis Region did not conduct rFDA/rMFDA until 2017. Only health posts with cases in January 2020 have a coverage estimate for that year, which represents January only. One health district with predominantly non-local transmission and previous reactive interventions was not considered in this study and is not shown.
